# Association Between Radiotherapy (±Chemotherapy) and the Severity of Low Anterior Resection Syndrome After Rectal Cancer Surgery: Does Radiotherapy Separate Risk?

**DOI:** 10.3390/medsci14020220

**Published:** 2026-04-29

**Authors:** Sorinel Lunca, Gabriel Mihail Dimofte, Ana Maria Musina, Cristian Ene Roata, Constantin Osman, Wee Liam Ong, Stefan Morarasu

**Affiliations:** 1 Grigore T. Popa University of Medicine and Pharmacy, 700115 Iasi, Romania; sorinel.lunca@umfiasi.ro (S.L.); ana-maria.musina@umfiasi.ro (A.M.M.); cristian.roata@umfiasi.ro (C.E.R.); stefan_morarasu@umfiasi.ro (S.M.); 2 2nd Department of Surgical Oncology, Regional Institute of Oncology, 700483 Iasi, Romania

**Keywords:** low anterior resection syndrome, rectal cancer, rectal surgery, radiotherapy, functional outcomes

## Abstract

**Background**: Low anterior resection syndrome (LARS) is a frequent survivorship problem after sphincter-preserving rectal cancer surgery. Pelvic radiotherapy (RT), often combined with chemotherapy, is frequently implicated in LARS development, but its apparent effect may be confounded by low tumor location and diversion. We evaluated whether RT (±chemotherapy) separates the risk of postoperative LARS severity—especially major LARS—beyond classical anatomic and pathway determinants. **Methods**: We conducted a single-centre observational cohort study of operated rectal cancer patients managed between 2013 and 2024, who completed the Romanian-validated LARS score by standardized telephone interview after restoration of bowel continuity (up to 18 months postoperatively). Outcomes were postoperative LARS score, LARS category, and major LARS. Comparisons were performed by RT status and by oncologic treatment pattern. Multivariable logistic regression assessed associations with major LARS, adjusting a priori for tumor location and diverting ileostomy; furthermore, extended sensitivity models incorporated technical/pathway variables. Discrimination was explored using 5-fold cross-validated ROC/AUC. Item-level LARS responses were analyzed to characterize symptom phenotype. **Results**: Overall, 182 patients were included (RT: 106; no RT: 76); 43.4% had LARS (minor 14.8%, major 28.6%). RT-treated patients had higher postoperative LARS scores (median 21 vs. 12; *p* = 0.002) and a higher prevalence of major LARS (35.8% vs. 18.4%; *p* = 0.012). Across treatment patterns, LARS severity was highest in RT + chemotherapy. Item-level analyses indicated that RT-associated differences were driven mainly by urgency and clustering domains. In adjusted models, RT was not independently associated with major LARS, whereas low tumor location and diverting ileostomy were strong predictors. Discrimination for major LARS was modest: AUC 0.561 for RT alone, 0.643 for location + ileostomy, and 0.654 for location + ileostomy + RT (5-fold cross-validation). **Conclusions**: RT is associated with worse unadjusted postoperative bowel dysfunction after rectal cancer surgery and is linked to urgency/clustering-dominant symptom patterns. However, in this cohort, the risk of major LARS was predominantly explained by tumor location and diversion rather than RT alone, supporting integrated risk stratification and early symptom-directed survivorship care.

## 1. Introduction

Low anterior resection syndrome (LARS) is among the most frequent and burdensome survivorship sequelae after sphincter-preserving rectal cancer surgery. It encompasses a constellation of bowel dysfunction symptoms (urgency, clustering/fragmentation of defecation, increased stool frequency, and fecal or flatus incontinence) that can persist for years and substantially impair quality of life, social functioning, and work productivity [1,2]. The syndrome has been operationalized in routine practice through the widely adopted LARS score, which enables pragmatic severity stratification, and more recently through an international consensus definition that broadens the focus from symptoms alone to include patient-important consequences and functional impact [3,4]. In large population-based and cohort studies, LARS severity correlates closely with patient-reported quality of life and functional outcomes, reinforcing that it is not merely a “benign postoperative problem” but a chronic functional disorder with meaningful survivorship implications [5,6]. Reported rates of major LARS vary widely across settings and follow-up windows, commonly in the range of 14–69%; however, major LARS is consistently associated with the poorest functional and quality-of-life profiles [7,8].

A consistent theme across literature is that LARS is multifactorial, arising from the interaction of anatomical constraints (tumor height and remnant rectal reservoir), surgical factors (extent of mesorectal excision and autonomic nerve injury), postoperative pathway elements (diversion and restoration), and oncologic therapy effects. Systematic reviews and meta-analyses repeatedly identify low tumor height, total mesorectal excision (TME), diverting stoma, and neoadjuvant radiotherapy (RT)/chemoradiotherapy as major predictors of severe/major LARS, although pooled estimates show substantial heterogeneity, reflecting differences in study design, timing of assessment, and case-mix [8,9,10]. Importantly, longitudinal evidence indicates that LARS is not static: many patients experience the highest symptom burden early after surgery and restoration of bowel continuity, with partial spontaneous improvement over months to years, while a clinically significant subgroup remains persistently symptomatic long-term [11,12,13]. This trajectory perspective emphasizes the need for early identification of high-risk patients and for distinguishing potentially modifiable pathway components (e.g., rehabilitation/structured management) from non-modifiable anatomic constraints [14].

Within this multifactorial framework, pelvic RT and chemoradiotherapy remain central and highly debated determinants. Mechanistically, irradiation may worsen anorectal function through mucosal injury, fibrosis and reduced compliance, microvascular damage, and neuromuscular effects on the sphincter complex and pelvic floor. These biologic pathways are consistent with clinical datasets associating neoadjuvant RT with higher postoperative LARS burden [9,15,16,17]. Evidence from randomized settings also supports the functional relevance of neoadjuvant treatment; for example, in a post hoc analysis of a randomized trial, long-course neoadjuvant radiation was associated with worse long-term bowel function and quality-of-life domains [15]. Complementing this, meta-analytic data specifically examining preoperative radiotherapy demonstrates adverse effects on long-term bowel function after anterior resection [9]. However, the interpretation of “RT as a risk factor” is complicated by treatment allocation and confounding by indication. RT is preferentially used in low and/or locally advanced tumors, which are themselves strongly associated with lower anastomoses, more extensive pelvic dissection (often TME), and higher likelihood of diversion, each independently increasing LARS risk [10,14,16,17,18,19]. This raises a clinically important question: in real-world cohorts, does RT independently separate the risk of the most severe phenotype (major LARS) once classic anatomic and pathway confounders are accounted for, or does it primarily act as a marker of a high-risk treatment trajectory?

In daily practice, RT exposure is frequently used as shorthand for “high risk of LARS” when counseling patients and planning follow-up, yet two gaps remain particularly relevant. First, many studies report overall LARS score or major-LARS prevalence without clarifying whether RT retains independent prognostic value beyond tumor location/height and diversion, which may dominate the risk structure. Second, fewer studies describe which symptom domains account for the apparent RT-associated effect, information that could support targeted management (e.g., urgency/clustering versus incontinence-dominant phenotypes). Against this background, we conducted a single-centre cohort study of operated rectal cancer patients assessed with the validated LARS score to evaluate whether radiotherapy (±chemotherapy) separates the risk of postoperative LARS severity, particularly major LARS, beyond tumor location and diverting ileostomy, and to explore whether a more granular oncologic treatment breakdown (RT + chemotherapy vs. chemotherapy alone vs. neither) improves interpretation of functional risk in routine practice.

## 2. Materials and Methods

### 2.1. Study Design and Setting

This investigation was designed as a single-centre observational cohort study including patients with rectal cancer treated at our institution between 2013 and 2024. All patients underwent standard oncologic staging and management according to institutional protocols. Indications for neoadjuvant radiotherapy were determined by a multidisciplinary tumor board based on preoperative staging, including tumor stage, nodal status, and circumferential resection margin involvement on MRI, rather than tumor location alone. All surgical procedures and subsequent follow-up were conducted at the same institution.

Low anterior resection syndrome (LARS) was assessed using the Romanian validated version of the LARS score, a five-item questionnaire endorsed by the European Society of Coloproctology. Data collection was performed through structured telephone interviews conducted by two investigators. This approach was chosen to ensure completeness of responses and allow real-time clarification of patient-reported outcomes. Patients who could not be contacted after repeated attempts were excluded from the analysis. A standardized script and a fixed sequence of questions were used to minimize interviewer-related variability.

Postoperative LARS evaluation was performed after restoration of intestinal continuity. In the present cohort, assessment occurred during postoperative follow-up within 18 months after surgery and/or after reversal of a diverting ileostomy when applicable. Patients who still had a diverting stoma at the time of the interview were not eligible for LARS evaluation.

Beyond the standard calculation of the total LARS score and its categorical classification, an item-level analysis of individual symptoms was also performed. For each question of the LARS questionnaire, the presence of a symptom was defined as an item score greater than zero, whereas maximum symptom severity corresponded to selection of the response option with the highest point allocation for that item. These item-specific variables were used to explore differences in symptom distribution according to radiotherapy exposure and across LARS severity categories (no LARS, minor LARS, and major LARS).

The study protocol was approved by the Institutional Ethics Committee. Due to the retrospective design of the study, the absence of patient risk, and the use of anonymized data, the requirement for individual informed consent was waived.

### 2.2. Eligibility Criteria

Eligible participants were patients who underwent low anterior resection (LAR) for rectal cancer and completed the LARS telephone questionnaire during follow-up. Patients were excluded if, at the time of the interview, they still had a diverting stoma with no restoration of bowel continuity, were unable to provide reliable responses due to impaired cognitive capacity, had died before the interview, or declined or failed to respond to the telephone contact. Patients managed non-operatively using a watch-and-wait strategy were not included in the analytical cohort.

### 2.3. Data and Statistical Analysis

Clinical and perioperative information was retrieved from the institutional electronic medical record system and compiled in a dedicated database (Microsoft Excel). Extracted variables included demographic characteristics (age and sex), perioperative parameters (operative approach, operative duration, and length of hospital stay), tumour-related variables (tumour location category and distance from the anal verge), operative details (presence of diverting stoma), oncologic treatments (radiotherapy and chemotherapy), and functional outcomes measured by the LARS score (total score, categorical classification, and responses to individual questionnaire items). The variable “chemotherapy” included both neoadjuvant (concurrent with radiotherapy) and adjuvant systemic treatment.

Continuous variables are reported as medians with interquartile ranges (IQRs), whereas categorical variables are presented as counts and percentages. Comparisons between groups were performed using the Mann–Whitney U test for continuous variables and Fisher’s exact test or the chi-square test for categorical variables, as appropriate. All statistical tests were two-tailed, and a *p*-value < 0.05 was considered statistically significant.

To identify factors associated with major LARS, univariable and multivariable logistic regression analyses were performed with major LARS as the dependent variable. The primary adjusted model incorporated radiotherapy exposure, tumour location, and the presence of a diverting ileostomy. Given the sample size and the distribution of key clinical variables, propensity score matching was not performed, as it would have resulted in a substantial reduction in the study population and statistical power. Instead, multivariable logistic regression models were used to adjust clinically relevant confounders, including tumor location and diverting ileostomy. Given the clinical interrelationship between variables, potential collinearity was checked using variance inflation factors (VIFs) for the variables included in the multivariable model. Additional models further included operative and technical variables such as the extent of mesorectal excision (TME vs. PME), anastomotic configuration or technique, and the surgical approach (open vs. robotic). Results are reported as odds ratios (ORs) with corresponding 95% confidence intervals (CIs).

The discriminative ability of the predictive models for major LARS was evaluated using receiver operating characteristic (ROC) curves and the corresponding area under the curve (AUC). To limit optimism bias in model performance estimates, ROC analysis was conducted using stratified five-fold cross-validation. Given the observational design, potential confounding by indication was anticipated, as radiotherapy is preferentially administered in patients with lower and more advanced tumors. To address this, multivariable logistic regression models were specified a priori to adjust for key clinical variables strongly associated with both treatment allocation and LARS risk, including tumor location and diverting ileostomy. These variables were selected based on clinical relevance and prior literature identifying them as dominant predictors of postoperative bowel dysfunction.

Missing data were handled using a complete-case strategy for each statistical model. Consequently, the effective sample size varies depending on the analysis performed and is reported alongside the respective model results in the tables.

All statistical analyses were carried out using XLSTAT version 2024.3 (Addinsoft, Paris, France) and MedCalc version 23.4.5.

## 3. Results

### 3.1. Study Population and LARS Overview

**Figure 1:**
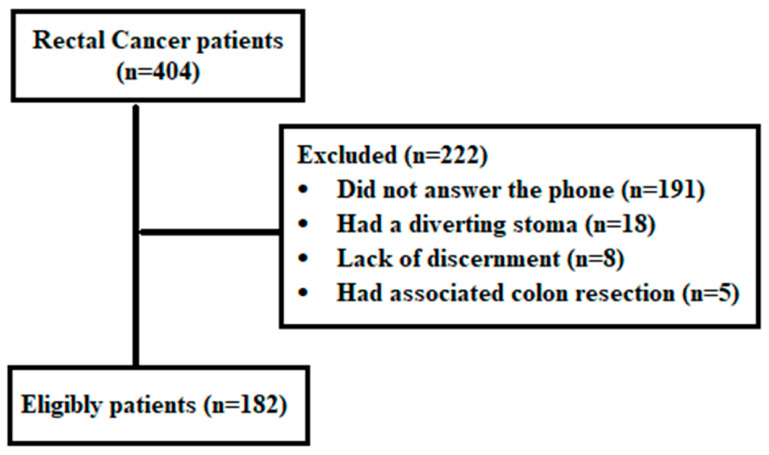
STROBE flowchart.

A total of 404 patients diagnosed with rectal cancer were identified through database screening and assessed for eligibility. After applying the predefined exclusion criteria—such as presence of a diverting stoma at the time of interview, impaired discernment, death, or failure to respond—182 patients who had undergone surgery and completed the LARS questionnaire were included in the final analysis (Figure 1).

Overall, postoperative bowel dysfunction was common. Major LARS was present in 52 patients (28.6%) and minor LARS in 27 patients (14.8%), corresponding to an overall LARS incidence of 43.4% (n = 79). The median postoperative LARS score in the cohort was 18 (IQR 9–30). (Table 1). Seventy patients (38.5%) reported receiving any treatment for bowel symptoms. Pharmacological therapy was the most commonly used approach in routine practice, primarily consisting of loperamide-based regimens, stool-bulking agents, or adsorbents, depending on the clinician’s preference.

### 3.2. Baseline Characteristics by Radiotherapy Status

Table 1 summarizes baseline and perioperative characteristics stratified by RT exposure. Of the 182 operated patients, 106 (58.2%) received RT and 76 (41.8%) did not. Median age was similar between groups (67 years in both; *p* = 0.462). Men were numerically more frequent in the RT group (60.4% vs. 46.1%), although this did not reach statistical significance (*p* = 0.070). Smoking was more prevalent among RT-treated patients (50.9% vs. 34.2%; *p* = 0.034), whereas BMI distributions were comparable (*p* = 0.589).

As expected, tumour position differed substantially by RT exposure (*p* < 0.001). RT-treated patients more frequently had mid- and low-rectal tumours (mid rectum: 67.0%; low rectum: 8.5%), whereas non-RT patients predominantly had upper-rectal tumours (69.7%). Consistently, the distance to the anal verge was shorter in the RT group (median 80 mm [IQR 68–100] vs. 100 mm [IQR 79–140]; *p* = 0.001).

RT exposure was also closely associated with treatment-pathway characteristics. Chemotherapy was more common in RT-treated patients (90.6% vs. 52.6%; *p* < 0.001), and diverting ileostomy was performed substantially more often in the RT group (84.0% vs. 35.5%; *p* < 0.001), highlighting the expected confounding-by-indication structure between oncologic therapy and anatomic/technical determinants of bowel function.

### 3.3. LARS Severity by Radiotherapy Status and Treatment Pattern

**Figure 2:**
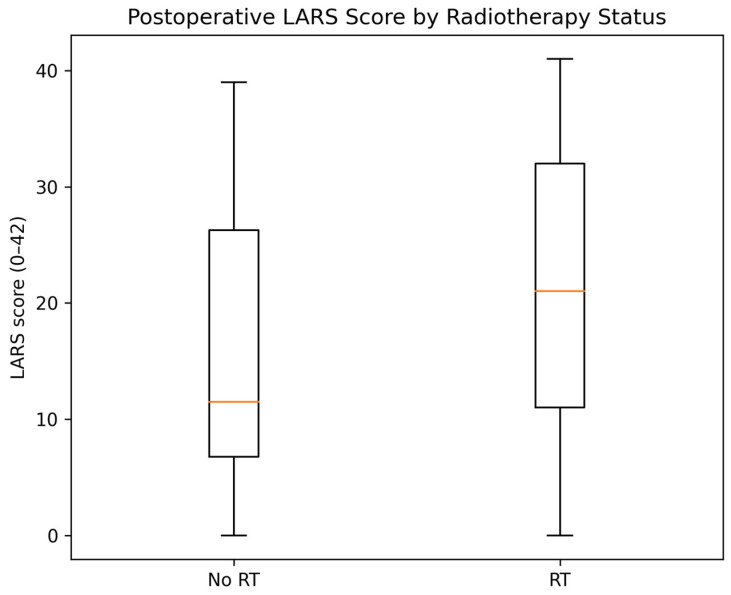
Postoperative LARS score according to radiotherapy status. Boxplots show the distribution of postoperative LARS scores in patients with and without radiotherapy. Horizontal orange lines represent medians, boxes indicate interquartile ranges, and whiskers denote range.

Postoperative LARS severity differed by RT exposure in unadjusted analyses (Table 2, Figure 2). Patients who received RT had higher postoperative LARS scores (median 21 [IQR 11–32] vs. 12 [IQR 7–26]; *p* = 0.002) and a higher proportion of major LARS (38/106, 35.8% vs. 14/76, 18.4%; *p* = 0.012). Across the three-category classification, RT-treated patients had fewer “No LARS” outcomes (49.1% vs. 67.1%) and more major LARS outcomes (35.8% vs. 18.4%) (overall distribution *p* = 0.026).

**Figure 3:**
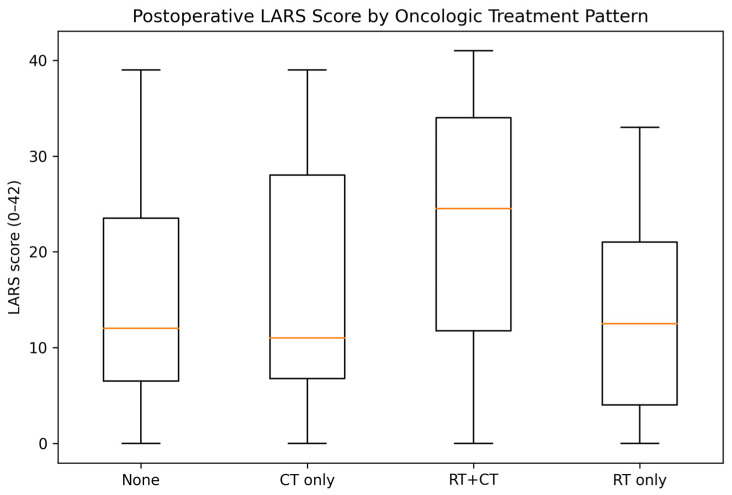
Postoperative LARS score according to oncologic treatment pattern. Boxplots display LARS scores stratified by treatment group (no oncologic treatment, chemotherapy only, radiotherapy plus chemotherapy, and radiotherapy only). Horizontal orange lines represent medians, boxes indicate interquartile ranges, and whiskers denote range.

A prespecified subgroup analysis compared common oncologic treatment patterns (Table 3, Figure 3). Median LARS scores were highest in patients treated with combined RT + chemotherapy (24 [IQR 12–34]) compared with chemotherapy alone (11 [IQR 7–28]) and no oncologic treatment (12 [IQR 6–24]) (overall *p* = 0.002). Major LARS prevalence also differed across treatment patterns (*p* = 0.046): 37.5% in RT + chemotherapy, 17.5% in chemotherapy-only, and 19.4% in the no-treatment group. The small “RT only” subgroup (n = 10) showed a median score of 12 [IQR 4–21] with 20.0% major LARS. The proportion of major LARS was similar between patients without combined oncological treatment and those receiving radiotherapy alone (19.4% vs. 20.0%), suggesting no clear unadjusted association between radiotherapy and functional outcomes.

Receipt of symptom-directed treatment did not differ significantly between RT strata (41.5% vs. 34.2%; *p* = 0.356), suggesting that the observed differences in symptom burden were not explained by differential treatment rates.

To assess whether RT independently separated the risk of major LARS after accounting for classical confounding, we fitted prespecified multivariable models (Table 4). In the primary adjusted model (RT + tumour location + ileostomy), RT was not independently associated with major LARS (OR 1.43, 95% CI 0.61–3.34; *p* = 0.414, VIF 1.405), whereas low tumour location (vs. upper rectum) (OR 7.16, 95% CI 1.82–28.16; *p* = 0.005, VIF 1.275) and ileostomy (OR 2.78, 95% CI 1.08–7.15; *p* = 0.033, VIF 1.435) were significant predictors. Assessment of multicollinearity using variance inflation factors showed no evidence of significant collinearity between predictors (all VIF values < 2), supporting the stability of the regression models.

**Figure 4:**
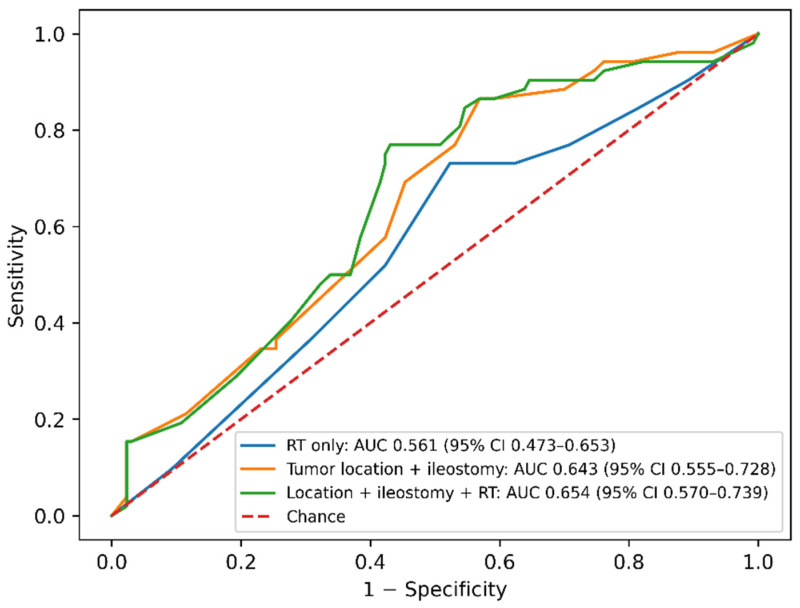
Cross-validated ROC curves for prediction of major LARS. Receiver operating characteristic (ROC) curves comparing predictive performance of different models using 5-fold cross-validation. Colours represent the different predictive models. The diagonal line indicates no discrimination (AUC = 0.50).

As shown in Figure 4, predictive discrimination for major LARS was limited when radiotherapy (RT) was used as a standalone predictor, with cross-validated AUC in the poor range (AUC 0.561, 95% CI 0.473–0.653). Incorporating classical anatomic/pathway determinants (tumor location and diverting ileostomy) improved discrimination to a modest level (AUC 0.643, 95% CI 0.555–0.728). Adding RT on top of tumor location and ileostomy produced only a small incremental gain in AUC, suggesting that most of the discrimination for major LARS in this cohort is explained by anatomic/pathway features rather than RT exposure alone (AUC 0.654, 95% CI 0.570–0.739). Cross-validated AUC estimates with bootstrap-derived 95% confidence intervals and standard errors are summarized in Table 5.

**Figure 5:**
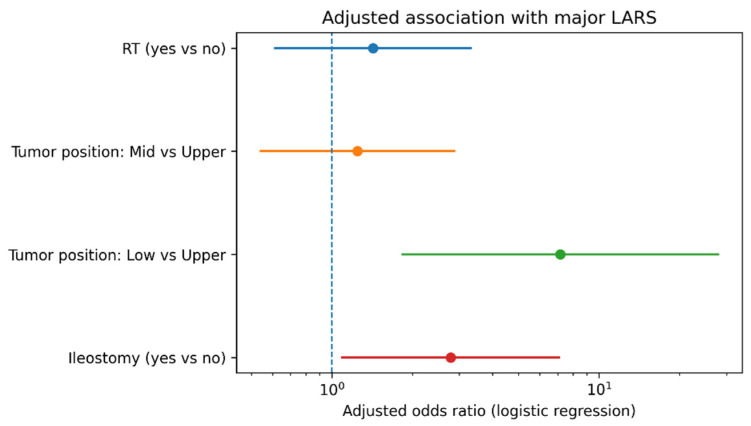
Forest plot of adjusted odds ratios for major LARS (log scale).

As shown in Figure 5, in the prespecified adjusted model (RT + tumor location + diverting ileostomy), low tumor location and diverting ileostomy were associated with higher odds of major LARS, whereas RT was not independently associated with major LARS after adjustment. The magnitude of association was greatest for low vs. upper rectal tumors, consistent with tumor height being a dominant driver of postoperative bowel dysfunction.

An extended technical/pathway model incorporating mesorectal excision type (TME/PME), anastomosis configuration (TT/LT), anastomosis technique (stapled/manual), and surgical approach (open/robotic) yielded consistent conclusions (Table 6). RT remained non-significant (OR 1.44, 95% CI 0.59–3.48; *p* = 0.420), while low tumour location (OR 9.33, 95% CI 2.00–43.45; *p* = 0.004) and ileostomy (OR 4.03, 95% CI 1.37–11.88; *p* = 0.011) retained associations with major LARS. Manual anastomosis was associated with higher odds of major LARS (stapled vs. manual OR 6.69, 95% CI (1.23–36.47; *p* = 0.028).

### 3.4. Supplementary Analyses: Symptom Phenotype, and Sensitivity Models

Item-level analyses clarified the symptom phenotype associated with RT exposure (Supplementary Table S1). Symptoms related to clustering (Q4) and urgency (Q5) were more frequent among RT-treated patients. For clustering, 57.5% of RT-treated patients reported any clustering (vs. 30.3% without RT; *p* < 0.001) and 42.5% reported maximum-severity clustering (vs. 18.4%; *p* = 0.001). For urgency, 63.2% of RT-treated patients reported any urgency (vs. 40.8%; *p* = 0.004). In contrast, flatus incontinence (Q1), liquid stool incontinence (Q2), and high stool frequency (Q3) did not differ materially by RT status in this cohort (all *p* > 0.10).

Symptom decomposition across LARS categories (Supplementary Table S2) showed that major LARS was characterized by near-universal clustering and urgency, while patients classified as “No LARS” could still report non-trivial symptoms, particularly flatus incontinence and increased stool frequency, underscoring that categorical cut-offs may underrepresent symptom burden at the individual level.

In sensitivity analysis using distance to anal verge as a continuous predictor (per 10 mm), RT remained non-significant (OR 1.62, 95% CI 0.71–3.73; *p* = 0.255) (Supplementary Table S3). Distance to anal verge was not independently associated with major LARS in this model (OR 1.02 per 10 mm, 95% CI 0.94–1.10; *p* = 0.711), whereas ileostomy remained associated (OR 4.06, 95% CI 1.40–11.75; *p* = 0.010). In this sensitivity model, robotic approach showed a modest association with major LARS compared with open surgery (OR 3.05, 95% CI 1.01–9.26; *p* = 0.049), noting the smaller robotic subgroup.

A restricted cubic spline sensitivity analysis for distance to anal verge did not provide evidence of non-linearity beyond the linear specification (likelihood ratio test *p* = 0.686; Supplementary Table S4), supporting the use of a simpler linear term in this dataset.

## 4. Discussion

In this single-centre cohort of rectal cancer survivors assessed with the validated LARS score, RT was associated with a higher postoperative symptom burden in unadjusted analyses, but did not remain an independent predictor of major LARS after adjustment for the classical anatomical/pathway determinants (tumor location and diverting ileostomy). The same pattern persisted across sensitivity analyses that incorporated operative covariates. These findings address a key practical issue in surgical counselling: whether RT alone serves as a dependable boundary between “low” and “high” risk of severe LARS, or whether its impact is better understood as part of a broader clinical pathway shaped by low tumour height, TME, diversion, and postoperative pelvic complications.

Our findings should be interpreted within the modern conceptualization of LARS as a survivorship syndrome defined not only by stool frequency or diarrhea, but by urgency, clustering, incontinence, and downstream functional impact. The LARS score was developed and validated as a pragmatic severity measure and has become the dominant tool used in contemporary outcome studies [3,4]. Importantly, an international consensus definition emphasized that “LARS” is best understood through both symptom burden and its consequences (“bother,” lifestyle restriction), reinforcing why studies must go beyond binary exposures and consider pathway interactions [1]. International guidance for management (MANUEL) further underscores that LARS is multifactorial and requires structured evaluation and stepwise interventions rather than attributing symptoms to a single cause such as RT [20]. Within this framework, the central contribution of our study is not to deny that RT matters, but to show that RT alone is an insufficient discriminator of severe LARS risk once anatomy and diversion are accounted for, an inference consistent with the broader evidence base.

In our cohort, RT exposure was associated with significantly higher postoperative LARS scores and a higher prevalence of major LARS in unadjusted analyses. This direction of association is consistent with population-based and randomized evidence, including systematic reviews/meta-analyses that repeatedly identify RT (often as part of chemoradiotherapy) among the strongest correlates of severe bowel dysfunction after sphincter-preserving surgery [8,9,10]. Croese et al. synthesized prevalence and risk factors and found RT to be a recurrent predictor across heterogeneous cohorts [8]. In a later meta-analysis, Sun et al. confirmed that neoadjuvant therapy (including RT/CRT) and low tumor height are among the most consistently reported predictors of LARS [10]. Liang et al. specifically evaluated preoperative RT effects on long-term bowel function and reported pooled evidence supporting worse LARS-related outcomes in irradiated patients [9]. Randomized and quasi-randomized data provide particularly persuasive support for a radiotherapy signal. The multicentre randomized follow-up report of short-course preoperative radiotherapy plus TME versus TME alone demonstrated worse bowel function many years after treatment in the irradiated arm, supporting the concept that RT can produce durable functional sequelae beyond the immediate postoperative phase [21]. The FOWARC post hoc randomized analysis similarly reported worse postoperative LARS and quality-of-life metrics after long-course neoadjuvant radiation strategies, reinforcing that RT can meaningfully influence postoperative bowel function even when surgery is standardized [15]. Cross-sectional observational studies have also shown higher bowel dysfunction rates after low anterior resection in patients treated with neoadjuvant therapy, including RT-containing regimens [22,23].

Despite this robust unadjusted association, RT did not remain independently significant in our adjusted model once tumor location and diverting ileostomy were included. This attenuation is not unexpected and is arguably the most clinically informative aspect of the analysis. These findings suggest that the apparent impact of radiotherapy on LARS observed in unadjusted analyses may be largely driven by underlying clinical and anatomical factors rather than treatment exposure itself.

RT is not randomly assigned in routine care. It is preferentially used for low-lying tumors and locally advanced disease, precisely those cases requiring more extensive pelvic dissection, lower anastomoses, and more frequent diversion. Tumor height is repeatedly shown as one of the most powerful drivers of major LARS across cohorts and meta-analyses [8,9,10]. Diversion has also been linked to worse long-term bowel dysfunction in multiple studies [24,25]. Thus, RT may be partly acting as a marker of the high-risk anatomical pathway rather than a standalone driver. The literature repeatedly emphasizes that LARS is multifactorial and that risk factors cluster; therefore, it is not surprising that a variable like RT, highly correlated with low tumor location and diversion, may lose statistical independence when those determinants are included [9,10,26].

This is consistent with the interpretation advanced in mechanistic and clinical reviews: the magnitude of RT’s effect is often difficult to separate from the effects of low anastomosis, TME extent, and pelvic floor disruption [26]. In practice, RT may “add weight” to a pathway already predisposed to severe dysfunction, but it may not be the dominant splitter of major-LARS risk once anatomy is known.

Low tumor location remained the strongest independent predictor of major LARS in our analysis. This is consistent with the “core” LARS risk literature. Meta-analyses repeatedly identify low tumor height/low anastomosis among the strongest predictors of severe LARS [8,9,10]. Large cohorts and trials similarly reinforce that tumor location and the consequences of low reconstruction dominate long-term bowel outcomes. For example, the international ROLARR trial reported substantial LARS incidence and emphasized that functional dysfunction is common even in modern standardized trial settings, in which tumor height and pelvic dissection remain key determinants irrespective of surgical platform [7]. Population-based analyses further show that LARS is common and meaningfully affects quality of life, reinforcing the importance of anatomical determinants in survivorship counselling and follow-up planning [27].

Longitudinal work demonstrates that LARS symptoms can persist or evolve over time, but severe dysfunction tends to remain concentrated among patients with low tumors and more extensive pelvic interventions [13,28]. Pieniowski et al. demonstrated that LARS is common and impacts quality of life, and that severe symptoms can persist in long-term follow-up [28]. Varghese et al., using an individual patient meta-analysis approach, highlighted trajectories over time and the persistence of severe dysfunction in substantial subsets, underscoring the need to identify high-risk patients early and not rely on a single exposure such as RT [13]. These studies collectively support our inference that tumor location provides more prognostic information about major LARS than RT alone.

Diverting ileostomy was an independent predictor of major LARS in our adjusted model. Observational data suggest that defunctioning stoma is associated with worse long-term bowel dysfunction and/or delayed functional adaptation after reversal [24]. A systematic review/meta-analysis specifically examining the impact of temporary ileostomy also supports the association between diversion and worse long-term functional outcomes in at least a subset of patients, though heterogeneity exists [25]. In addition, diversion is often correlated with low anastomosis and postoperative complications (including pelvic sepsis), which themselves worsen long-term function—further reinforcing the pathway-coupling concept [24,25].

In addition, manual (hand-sewn) anastomosis was associated with increased odds of major LARS compared with stapled anastomosis. This finding warrants careful interpretation. In contemporary rectal cancer surgery, hand-sewn anastomosis is typically performed in technically demanding situations, particularly in very low rectal tumors or in patients with unfavorable pelvic anatomy, where the use of a circular stapler may be limited or not feasible. These conditions are independently associated with a higher risk of postoperative bowel dysfunction, owing to factors such as shorter residual rectal length, reduced neorectal reservoir capacity, and increased risk of pelvic autonomic nerve disturbance during deep pelvic dissection. Consequently, the observed association between manual anastomosis and major LARS is likely to reflect underlying anatomical and technical complexity rather than a direct effect of the anastomotic technique itself. Furthermore, the wide confidence interval observed in our model suggests limited precision, and this finding should therefore be considered hypothesis-generating.

Our prespecified treatment pattern subanalysis (RT + CT vs. CT-only vs. none) showed the highest symptom burden and highest major LARS prevalence in the combined RT + CT group, while CT-only and no-neoadjuvant groups were similar. This observation mirrors literature differentiating CRT and non-RT strategies. Cross-sectional cohorts comparing CRT versus chemotherapy alone reported worse bowel dysfunction in RT-containing regimens, even when accounting for surgical approach and staging differences [23]. A population-based cross-sectional study also reported worse bowel function patterns in patients exposed to neoadjuvant therapy, including RT/CRT [22]. Moreover, modern trial datasets and survivorship analyses highlight bowel function as a key toxicity endpoint in contemporary neoadjuvant strategies [29,30].

This is highly relevant in the era of de-escalation and selective radiotherapy. PROSPECT demonstrated that neoadjuvant chemotherapy with selective RT can achieve oncologic outcomes comparable to standard CRT in selected intermediate-risk patients, bringing renewed attention to functional preservation and patient-reported outcomes [31]. PROSPECT patient-reported outcomes analyses further emphasize differential symptom burdens across treatment strategies, underscoring the need to incorporate bowel function into decision-making rather than focusing solely on local control metrics [32]. Our real-world cohort’s pattern—RT + CT being worse and CT-only similar to none—provides additional pragmatic support that, where oncologically safe, minimizing RT exposure may reduce long-term bowel dysfunction risk in some patients.

TNT strategies such as RAPIDO, while oncologically impactful, also raise functional questions because intensified neoadjuvant sequences may affect pelvic organ function and quality of life. RAPIDO quality-of-life and late toxicity analyses highlight that bowel toxicity remains a key survivorship issue in intensified regimens [29]. Likewise, interval or “delay” strategies after short-course RT have reported clinically relevant toxicity during the neoadjuvant interval, reminding clinicians that functional burden can appear both early and late [33]. These trial-based data support the broader inference: radiotherapy sequencing and selection may shape functional outcomes in complex ways that extend beyond the binary “RT yes/no” label [29,30,31,32,33].

Radiotherapy may influence not only severity but also chronicity of symptoms; randomized long-term follow-up supports durable RT-associated bowel dysfunction [21], and trial-embedded toxicity reporting suggests that RT-related functional toxicity can appear both during neoadjuvant intervals and long after surgery [29,33]. Thus, the question “does RT separate risk?” may have time-dependent answers: RT may contribute to persistent urgency/clustering symptoms, while the binary major-LARS phenotype remains strongly structured by anatomy and diversion [13,21,26,29,33,34].

It is also important to consider that modern radiotherapy strategies may modify the functional impact of treatment. Advances in conformal techniques such as IMRT and VMAT have been shown to reduce radiation dose to the anal sphincter complex compared with conventional 3D radiotherapy [35]. In addition, systematic reviews of dose–volume effects emphasize the importance of minimizing dose to anorectal structures, with suggested constraints such as anal canal Dmean < 40 Gy to reduce late toxicity [36]. Emerging evidence suggests that such approaches may decrease long-term bowel toxicity, although their specific impact on LARS remains to be fully defined [31,34,35,36,37].

Our cross-validated ROC analyses indicated that RT alone had limited discrimination for major LARS, whereas models incorporating tumor location and diversion achieved moderate discrimination; furthermore, adding RT yielded only incremental improvement. This is consistent with the concept that RT is an important contributor to symptom burden but a weak standalone classifier of the severe phenotype once anatomy is known. The literature increasingly emphasizes model-based risk stratification, including post-RT prediction modeling efforts specifically for major LARS [38] and risk factor synthesis from surgical cohorts [39,40,41]. However, even advanced models tend to show only moderate AUCs, consistent with LARS being influenced by unmeasured factors (baseline bowel function, pelvic floor reserve, psychosocial adaptation, adherence to rehabilitation) and by postoperative events not fully captured in routine datasets [26]. Although radiotherapy, tumor location, and diverting ileostomy are closely related within the treatment pathway, the stability and consistency of the multivariable model suggest that collinearity did not materially influence the results.

From a clinical standpoint, our findings reinforce two messages relevant to multidisciplinary rectal cancer care. First, RT exposure alone is an insufficient stand-alone marker of severe postoperative bowel dysfunction; risk communication and follow-up planning should integrate tumour height, diversion strategy, and technical factors, which were the strongest predictors in our models. Second, because urgency and clustering appear to be the domains most linked to RT in our cohort, early screening and symptom-directed interventions for these components may be particularly valuable in RT-treated patients.

Several limitations should be acknowledged. A key limitation of the present study is the potential for confounding by indication, inherent to observational analyses of radiotherapy in rectal cancer. Patients receiving radiotherapy more frequently had mid- and low-rectal tumors and underwent diverting ileostomy, both of which are strongly associated with LARS. Although multivariable adjustment was performed, residual confounding cannot be fully excluded. Importantly, the attenuation of the association between radiotherapy and LARS after adjustment suggests that radiotherapy may act primarily as a marker of a higher-risk clinical pathway rather than an independent determinant.

The present study is also subject to potential selection bias, as a substantial proportion of eligible patients were excluded due to inability to establish telephone contact. Although telephone-based assessment ensured data completeness, patients who could not be reached may differ systematically, limiting generalizability.

Although propensity score methods may improve comparability between treatment groups, their application in this cohort would likely have resulted in loss of statistical power. Future studies with larger populations may benefit from such approaches.

A proportion of patients with upper rectal tumors received radiotherapy, reflecting individualized multidisciplinary decision-making based on staging and MRI features. While this introduces heterogeneity, it reflects real-world clinical practice.

Additional limitations include the lack of detailed dosimetry data, variability in RT techniques, and the absence of longitudinal symptom trajectories beyond 18 months.

## 5. Conclusions

In conclusion, our results confirm that radiotherapy is associated with increased postoperative bowel dysfunction in unadjusted comparisons. After accounting for tumor location and diversion, RT did not independently separate the risk of major LARS in our cohort. This supports a clinically pragmatic interpretation: radiotherapy is better understood as a contributing modifier within a multifactorial pathway driven primarily by anatomy, reconstruction, diversion, and postoperative pelvic events, rather than as an independent determinant of severe LARS risk.

## Figures and Tables

**Table 1:** Baseline clinical, pathological, and treatment characteristics according to radiotherapy exposure.

Variable	No RT (n = 76)	RT (n = 106)	*p* Value
Age, years	67 [60–73]	67 [58–71]	0.462
Male sex	35 (46.1%)	64 (60.4%)	0.070
BMI, kg/m^2^	27.5 [24.5–29.7]	28.0 [24.8–30.5]	0.589
Diabetes	13 (17.1%)	18 (17.0%)	1.000
Frail	35 (46.1%)	42 (39.6%)	0.447
Smoking	26 (34.2%)	54 (50.9%)	0.034
Tumor location, n (%)			<0.001
Upper rectum	53 (69.7%)	26 (24.5%)	
Mid rectum	19 (25.0%)	71 (67.0%)	
Low rectum	4 (5.3%)	9 (8.5%)	
Distance to anal verge, mm	63 [0–106]	78 [51–96]	0.110
(y)pT stage, n (%)			
pT0–1	12 (17.9%)	25 (22.5%)	
pT2	14 (20.9%)	28 (25.2%)	
pT3	35 (52.2%)	56 (50.5%)	
pT4	6 (9.0%)	2 (1.8%)	
(y)pN stage, n (%)			
N0	42 (62.7%)	76 (68.5%)	
N1–2	25 (37.3%)	35 (31.5%)	
Total mesorectal excision	40 (52.6%)	93 (87.7%)	<0.001
Diverting ileostomy	27 (35.5%)	89 (84.0%)	<0.001
Surgical approach, n%			0.048
Open	60 (78.9%)	89 (84.0%)	
Robotic	16 (21.1%)	10 (9.4%)	
Time to reversal, days	56 [42–99]	57 [42–102]	0.632
Operative time, min	202 [154–258]	210 [175–260]	0.597
Length of stay, days	8 [6–11]	8 [7–10]	0.691
Chemotherapy †	40 (52.6%)	96 (90.6%)	<0.001
Follow-up, months	27.3 [13.2–65.6]	29.8 [14.3–66.7]	0.435

Note: RT, radiotherapy; BMI, body mass index. Pathological staging was reported according to the TNM classification, using the prefix “y” for patients who received neoadjuvant therapy. Percentages are calculated based on available cases. † Chemotherapy includes both neoadjuvant (concurrent with radiotherapy) and adjuvant systemic treatment.

**Table 2:** LARS outcomes by radiotherapy status.

Outcome	No RT (n = 76)	RT (n = 106)	*p* Value
LARS score	12 [7–26]	21 [11–32]	0.002
LARS category, n (%)			0.026
No LARS	51 (67.1%)	52 (49.1%)	
Minor LARS	11 (14.5%)	16 (15.1%)	
Major LARS	14 (18.4%)	38 (35.8%)	
Major LARS (binary)	14 (18.4%)	38 (35.8%)	0.012

Key: RT, radiotherapy; LARS, low anterior resection syndrome.

**Table 3:** LARS severity according to oncologic treatment pattern.

Oncologic Treatment Group	n	Median LARS Score (IQR)	Major LARS, n (%)
None	36	12 [6–24]	19.4%
Chemotherapy only (CT)	40	11 [7–28]	17.5%
Radiotherapy + Chemotherapy (RT + CT)	96	24 [12–34]	37.5%
Radiotherapy only (RT)	10	12 [4–21]	20.0%

Key: LARS, low anterior resection syndrome; IQR, interquartile range.

**Table 4:** Multivariable logistic regression for major LARS (adjusted for tumor location and diverting ileostomy).

Predictor	Adjusted OR (95% CI)	*p* Value
Radiotherapy (yes vs. no)	1.43 (0.61–3.34)	0.414
Tumor location: Mid vs. Upper	1.25 (0.54–2.89)	0.610
Tumor location: Low vs. Upper	7.16 (1.82–28.16)	0.005
Diverting ileostomy (yes vs. no)	2.78 (1.08–7.15)	0.033

**Key:** OR, odds ratio.

**Table 5:** Five-fold cross-validated ROC performance for predicting major LARS.

Predictors	AUC	SE	95% CI
RT only	0.561	0.046	0.473–0.653
Tumor location + diverting ileostomy	0.643	0.044	0.555–0.728
Tumor location + diverting ileostomy + RT	0.654	0.044	0.570–0.739

Key: AUC, area under the curve; SE, standard error; CI, confidence interval. AUCs were computed using five-fold stratified cross-validation with out-of-fold predicted probabilities. Confidence intervals and SEs were obtained via bootstrap resampling (4000 iterations) of the out-of-fold predictions.

**Table 6:** Multivariable logistic regression for major LARS including surgical technique variable.

Predictor	Adjusted OR (95% CI)	*p* Value
Radiotherapy (yes vs. no)	1.44 (0.59–3.48)	0.421
Tumor location: Mid vs. Upper	1.81 (0.64–5.16)	0.264
Tumor location: Low vs. Upper	9.33 (2.00–43.47)	0.004
Diverting ileostomy (yes vs. no)	4.03 (1.37–11.88)	0.011
Mesorectal excision: PME vs. TME	1.81 (0.56–5.90)	0.322
Anastomosis configuration: LT vs. TT	1.03 (0.46–2.33)	0.936
Anastomosis technique: Manual vs. Stapled	6.69 (1.23–36.47)	0.028
Approach: Robotic vs. Open	2.29 (0.70–7.49)	0.169

**Key:** OR, odds ratio; PME, partial mesorectal excision; TME, total mesorectal excision; LT, lateroterminal (i.e., side to end); TT, termino-terminal (i.e., end to end).

## Data Availability

The original contributions presented in this study are included in the article/Supplementary Materials. Further inquiries can be directed to the corresponding author.

## Supplementary Materials

The following supporting information can be downloaded at: https://www.mdpi.com/article/10.3390/medsci14020220/s1, Table S1: LARS items by RT status: median [IQR], prevalence of any symptom (item score > 0), and prevalence of maximum-severity response; Table S2: LARS items stratified by LARS category (No/Minor/Major): any symptom, maximum severity response, and median [IQR]; Table S3: Multivariable logistic regression for major LARS including RT, distance to anal verge (continuous), ileostomy, mesorectal excision extent (TME/PME), anastomosis configuration and technique, and surgical approach; Table S4: Multivariable logistic regression for major LARS with a restricted cubic spline term for distance to anal verge, adjusted for RT, ileostomy, mesorectal excision extent (TME/PME), anastomosis configuration and technique, and surgical approach. The spline term is reported with an overall Wald *p*-value; illustrative adjusted ORs compare selected distances at a fixed reference profile.

## Abbreviations

The following abbreviations are used in this manuscript:

AUC	Area under the curve
BMI	Body mass index
CI	Confidence interval
CRT	Chemoradiotherapy
CT	Chemotherapy
CV	Cross-validation
IMRT	Intensity-Modulated Radiation Therapy
LAR	Low anterior resection
LARS	Low anterior resection syndrome
OR	Odds ratio
PME	Partial mesorectal excision
ROC	Receiver operating characteristic
RT	Radiotherapy
SE	Standard error
TME	Total mesorectal excision
VAMT	Volumetric Modulated Arc Therapy
