# Supplementary material for: Association Between Radiotherapy (±Chemotherapy) and the Severity of Low Anterior Resection Syndrome After Rectal Cancer Surgery: Does Radiotherapy Separate Risk?
Source: Med Sci (Basel). 2026 Apr 29;14(2):220. doi: 10.3390/medsci14020220 (PMC13214608; doi:10.3390/medsci14020220)
Supplement: Supplementary file 1 [file medsci-14-00220-s001.zip › medsci-4223855-supplementary.pdf]

Supplementary Materials

Table S1. LARS items by RT status: median [IQR], prevalence of any symptom (item score > 0), and prevalence of maximum-severity response.

Item	No RT median [IQR] (n=76)	RT median [IQR] (n=106)	Any symptom (No RT)	Any symptom (RT)	p (any)	Max severity (No RT)	Max severity (RT)	p (max)	Max score value
**LARS Q1**	7 [0–7]	4 [0–7]	49 (64.5%)	72 (67.9%)	0.637	40 (52.6%)	47 (44.3%)	0.295	7
**LARS Q2**	0 [0–3]	0 [0–3]	25 (32.9%)	37 (34.9%)	0.874	25 (32.9%)	37 (34.9%)	0.874	3
**LARS Q3**	2 [0–2]	2 [0–4]	46 (60.5%)	65 (61.3%)	1.000	15 (19.7%)	16 (15.1%)	0.430	5
**LARS Q4**	0 [0–9]	9 [0–11]	23 (30.3%)	61 (57.5%)	<0.001	14 (18.4%)	45 (42.5%)	<0.001	11
**LARS Q5**	0 [0–16]	11 [0–16]	31 (40.8%)	67 (63.2%)	0.004	21 (27.6%)	36 (34.0%)	0.419	16

Table S2. LARS items stratified by LARS category (No/Minor/Major): any symptom, maximum severity response, and median [IQR].

Item	LARS category	Any symptom	Max severity	Median [IQR]
**LARS Q1**	No LARS	55 (53.4%)	33 (32.0%)	4 [0–7]
**LARS Q1**	Minor LARS	19 (70.4%)	17 (63.0%)	7 [0–7]
**LARS Q1**	Major LARS	47 (90.4%)	37 (71.2%)	7 [4–7]
**LARS Q2**	No LARS	19 (18.4%)	19 (18.4%)	0 [0–0]
**LARS Q2**	Minor LARS	7 (25.9%)	7 (25.9%)	0 [0–2]
**LARS Q2**	Major LARS	36 (69.2%)	36 (69.2%)	3 [0–3]
**LARS Q3**	No LARS	54 (52.4%)	22 (21.4%)	2 [0–2]
**LARS Q3**	Minor LARS	19 (70.4%)	7 (25.9%)	2 [0–4]
**LARS Q3**	Major LARS	38 (73.1%)	2 (3.8%)	2 [0–3]
**LARS Q4**	No LARS	20 (19.4%)	9 (8.7%)	0 [0–0]
**LARS Q4**	Minor LARS	13 (48.1%)	8 (29.6%)	0 [0–11]
**LARS Q4**	Major LARS	51 (98.1%)	42 (80.8%)	11 [11–11]
**LARS Q5**	No LARS	20 (19.4%)	2 (1.9%)	0 [0–0]
**LARS Q5**	Minor LARS	27 (100.0%)	14 (51.9%)	16 [11–16]
**LARS Q5**	Major LARS	51 (98.1%)	41 (78.8%)	16 [16–16]

Table S3. Multivariable logistic regression for major LARS including RT, distance to anal verge (continuous), ileostomy, mesorectal excision extent (TME/PME), anastomosis configuration and technique, and surgical approach.

Predictor	Adjusted OR (95% CI)	p value
**Radiotherapy (yes vs no)**	1.62 (0.71–3.73)	0.255
**Distance to anal verge (per 10 mm increase)**	1.02 (0.94–1.10)	0.711
**Diverting ileostomy (yes vs no)**	4.06 (1.40–11.76)	0.010
**Mesorectal excision: PME vs TME**	1.01 (0.38–2.67)	0.992
**Anastomosis configuration: LT vs TT**	1.02 (0.46–2.25)	0.956
**Anastomosis technique: Manual vs Stapled**	5.59 (1.03–30.49)	0.047
**Approach: Robotic vs Open**	3.05 (1.01–9.26)	0.049

Table S4. Multivariable logistic regression for major LARS with a restricted cubic spline term for distance to anal verge, adjusted for RT, ileostomy, mesorectal excision extent (TME/PME), anastomosis configuration and technique, and surgical approach. The spline term is reported with an overall Wald p-value; illustrative adjusted ORs compare selected distances at a fixed reference profile.

Predictor	Adjusted effect	p value
**Distance to anal verge (restricted cubic spline, df=4)**	—	
**Adjusted OR for DAV 50 vs 100 mm (reference profile: RT=0, no ileostomy, Open, TME, TT, Stapled)**	0.94 (0.35–2.50)	
**Adjusted OR for DAV 70 vs 100 mm (same reference profile)**	1.01 (0.55–1.85)	
**Radiotherapy (yes vs no)**	1.45 (0.61–3.44)	0.401
**Diverting ileostomy (yes vs no)**	3.98 (1.35–11.76)	0.012
**Mesorectal excision: PME vs TME**	1.14 (0.41–3.19)	0.801
**Anastomosis configuration: LT vs TT**	0.99 (0.45–2.20)	0.986
**Anastomosis technique: Manual vs Stapled**	6.16 (1.08–35.21)	0.041
**Approach: Robotic vs Open**	3.08 (1.00–9.46)	0.050
